# Telebehavioral Health, In-Person, and Hybrid Modalities of Treatment Delivery Among US Service Members: Longitudinal Observational Study

**DOI:** 10.2196/83809

**Published:** 2026-01-05

**Authors:** Kristen H Walter, Lisa H Glassman, Jordan A Levine, Hee-Jin Jun, James F Bonkowski, Samuel Y Chung, Emily A Schmied

**Affiliations:** 1 Naval Health Research Center, 140 Sylvester Road, San Diego, CA, 92106, United States, 1 619-540-4108; 2 Leidos, Inc, Naval Health Research Center, San Diego, CA, United States; 3 School of Public Health, San Diego State University, San Diego, CA, United States

**Keywords:** telemedicine, teletherapy, mHealth, mobile health, eHealth, digital health, military, mental health, psychotherapy, therapy

## Abstract

**Background:**

The availability of telebehavioral health care dramatically increased in response to the COVID-19 pandemic among both civilian and military populations. After the restrictions were lifted, telebehavioral health use decreased but remained elevated compared to before the pandemic. Examining the use of treatment modalities and how they relate to care metrics can inform the future delivery of behavioral health care.

**Objective:**

This study aimed to explore behavioral health use patterns by treatment modality (telehealth, in-person, and hybrid care) among active duty service members with at least 1 of 12 behavioral health conditions. Treatment modality groups were also compared on the number of visits and between-visit intervals to determine the association with care metrics.

**Methods:**

The study included 588,928 active duty service members who completed at least 6 months of continuous service during the study period (October 1, 2015, to September 30, 2021) and received care for at least 1 behavioral health condition of interest. Personnel and demographic data were matched with medical reimbursement records. Diagnostic and treatment procedure codes were extracted for each health care visit. For each service member in the study population, the total number of behavioral health visits, modality of each visit, and average duration of time between visits were calculated.

**Results:**

Overall, 59.57% (350,843/588,928) of service members received only in-person care during the 6-year study period, 4.12% (24,245/588,928) received only telehealth, and 36.31% (213,840/588,928) received hybrid care. For 8 (66.7%) of the 12 behavioral health conditions (eg, alcohol use disorder, attention-deficit/hyperactivity disorder, generalized anxiety disorder, major depressive disorder, panic disorder, posttraumatic stress disorder, substance use disorder, and suicidal behavior), service members were more likely to receive hybrid care, whereas the other 4 (41.7%) conditions (eg, acute stress disorder, adjustment disorder, insomnia, and suicidal ideation) were more likely to be associated with in-person care. Service members who received hybrid care averaged 8 times more visits than those using only telehealth and 3 times more visits than those receiving only in-person care. For most conditions, service members who received in-person care only averaged the longest intervals between visits, whereas those who used telehealth care only averaged the shortest intervals. Among specific behavioral health conditions, average intervals were longest among those with attention-deficit/hyperactivity disorder, acute stress disorder, and insomnia (79‐89 d) and shortest among those with suicidal behavior, substance use disorder, and alcohol use disorder (25‐38 d).

**Conclusions:**

Telebehavioral health care was commonly used in combination with in-person care and associated with more health care visits and the least amount of time between visits, revealing advantages of offering telehealth within the Military Health System. Findings support a flexible care delivery approach that includes various modalities, such as telehealth, in-person, and hybrid options to address the behavioral health needs of service members.

## Introduction

### Background

Behavioral health care delivered via telehealth is not a new practice in the United States; however, the COVID-19 pandemic led to a rapid increase in the capability and accessibility of this treatment modality among both civilian and military populations. Telehealth is broadly defined by the American Telemedicine Association as a “mode of delivering healthcare services through the use of telecommunications technologies ...by a healthcare practitioner to a patient at a different physical location than the healthcare practitioner” [1], and the term has been used interchangeably with telemedicine, teletherapy, mobile health, eHealth, and digital health delivery across the literature. Telebehavioral health served a critical need during the pandemic, especially as nationally representative civilian data [2] showed dramatic increases in behavioral health symptoms and distress resulting from the pandemic. Among military populations across 5 countries, resiliency was demonstrated early in the pandemic. However, mental health worsened, and stress levels increased over time for certain subpopulations, such as service members who were deployed to provide aid and assistance in response to COVID-19 [3]. Telehealth provided a way to manage behavioral health needs across civilian and military populations during the pandemic by maintaining social distancing requirements and addressing gaps in care delivery. These characteristics support the ongoing and increased availability of this treatment modality.

For telehealth during the COVID-19 pandemic more generally, nationally representative civilian data showed that rates of telehealth visits increased by 17% during the first 6 calendar months of 2020 (from 0.8 to 17.8 visits per 1000 enrollees), whereas in-person visits decreased 26% (from 102.7 to 76.3 per 1000 enrollees) [4]. The increase in telehealth use was also evident among active duty service members, as telehealth visits increased by 20-fold, with 2,891,865 visits in 2020 compared to 138,138 in 2019 [5]. For behavioral health conditions more specifically, telehealth visits were approximately 25% higher during March through September 2020 compared with the same period in 2019 [6]. Rates of telebehavioral health visits among active duty service members peaked in April 2020 and declined by mid-June [6-8], a trend also observed across 11 behavioral health conditions [7]. Clark et al [6] further observed that military telebehavioral health visit rates stabilized after June 2020 but were consistently elevated compared with the prior year, implying that telehealth had become a larger part of the health care landscape and an option more readily available than before the pandemic. However, less is known about how the use of telebehavioral health fits within service members’ overall treatment use.

Although telehealth rates increased dramatically at the start of the pandemic, telehealth use had steadily increased over the prior decade [9,10]. Telehealth offers solutions to several barriers to in-person behavioral health care for patients, providers, and health care systems. For example, across patient populations, telebehavioral health can eliminate geographic constraints by delivering care to patients in remote and rural areas and those with provider shortages [11-15]. In the military, service members in austere, far-forward, and shipboard locations can receive telebehavioral health care, potentially reducing resource- and cost-intensive medical evacuations [16], for which behavioral health conditions are a leading cause [17,18]. Telebehavioral health also accommodates patients who prefer to receive care in their own home due to concerns related to mobility or to privacy and/or psychological comfort [11,12,19]. A private setting of the patient’s choosing may facilitate care seeking among those who otherwise would not receive care in a traditional medical facility due to perceived stigma—a barrier for many populations, especially the military [12,20-25]. These potential advantages of telehealth may also vary within military contexts. For example, an officer receiving behavioral health care on base may benefit from the privacy of telehealth, whereas a junior-enlisted service member living in barracks or quarters may have difficulty finding a private space to attend telehealth sessions. The options of telehealth and other delivery modalities allow service members the opportunity to access behavioral health care in ways that may best address their treatment needs.

Importantly, evidence suggests that psychotherapies delivered via telehealth are generally as effective at reducing behavioral health symptoms as in-person treatment in both civilian and military populations [22,26-28]. Furthermore, many patients receiving telebehavioral health reported similar relationship building with their therapist, comparable to in-person treatment [29,30]. For providers, telebehavioral health can result in increased clinical efficiency by reducing time to care initiation [31], shortening care episodes [32], and facilitating faster appointments and decreasing the frequency of no-shows [33]. Both patients and providers may experience a reduction in treatment-related expenses due to lower costs associated with travel, transportation, time, and missed work [19,34-37]. Health systems also benefit through reduced use of medical supplies, lower facility fees, and lower overhead costs [38]. In the Military Health System (MHS), the opportunity cost savings of telebehavioral health were determined to be over US $1.1 million for officers and US $740,000 for enlisted service members compared with in-person visits in 2020 [31]. Taken together, telebehavioral health provides numerous advantages for patients, providers, and health care systems and overcomes barriers associated with distance, preference, and cost.

Although telebehavioral health offers many benefits for care delivery, there are challenges, and it may not be suited for all patients. A main concern involves technology, such as competence with technology, comfort communicating over video conferencing, internet quality and connectivity, experience with software, and hardware that can restrict optimal performance [11,14,15,24,36,37,39]. Technology access issues may also exist in the very military settings where there is a critical need for telehealth care—austere, far forward, and shipboard locations. Socioeconomic factors affecting technological access and familiarity could also create a situation of *digital exclusion* from telehealth [40,41], leading to health care disparities. For example, while telehealth can promote patient privacy and comfort, some may lack a safe or private space, experience disruptions in their environment [11,14,42], or may not be able to afford fast connection speeds or updated devices that support telehealth use [43]. Additionally, although research generally shows comparable outcomes for telehealth and in-person psychotherapy [22,26-28], there are clinical subgroups that appear to benefit more from in-person treatment. Specifically, treatments for depression showed better outcomes when delivered in person than through telehealth [44]. Other subgroups with higher hopelessness or anxiety symptom severity also showed better outcomes for in-person care versus telehealth [26], and individuals with greater symptom severity and behavioral health comorbidities may be better matched to in-person treatment [45,46]. Given the differences between telebehavioral health and in-person treatment, it is important to understand the use of these modalities over time, including when used in combination.

### Objectives

Most existing research examining the expansion of telehealth use in response to the pandemic either assessed telehealth alone or changes in telehealth and in-person care as single modalities [4-6,32]; limited research has explored the combined use of telehealth and in-person care (ie, “hybrid” care). An exception is a recent RAND study conducted by Hepner et al [8], who reported that most service members who began behavioral health treatment in the early months of the pandemic received a hybrid of telehealth and in-person visits. This study examined the corresponding 6-month periods in 2019 and 2020 and focused on 3 diagnoses (ie, posttraumatic stress disorder [PTSD], depression, and substance use disorder [SUD]).

This study built on these findings by evaluating behavioral health use patterns by treatment modality (ie, telehealth, in-person, and hybrid care) among active duty service members with at least one of the 12 behavioral health diagnoses of interest over a 6-year period that extended to September 2021. Furthermore, the modalities were compared on the number of visits and between-visit intervals to determine whether the modalities were associated with care metrics. Study results can inform the future delivery of behavioral health care to service members, supporting the aims of the Department of Defense [47] and the Defense Health Agency [48]. On a broader level, this study raises considerations for flexible delivery, personal choice, and shared decision-making in behavioral health care [22,49,50].

## Methods

### Data Sources

The base population consisted of active duty military service members with at least 6 months of continuous service during the study period (October 1, 2015, to September 30, 2021) and who received care for at least 1 behavioral health condition of interest. The study period began in 2015, as telehealth care was seldom used in the MHS before this time [7,51,52]. The 12 behavioral health conditions of interest included acute stress disorder (ASD), adjustment disorder, alcohol use disorder (AUD), attention-deficit/hyperactivity disorder (ADHD), generalized anxiety disorder (GAD), insomnia, major depressive disorder (MDD), suicidal behavior, panic disorder, PTSD, SUD, and suicidal ideation. These selected behavioral health conditions are common in military populations and have been explored in prior research using similar data sources [7,53].

Personnel and demographic data were derived from the Career History Archival Medical and Personnel System and then matched with specific diagnoses from medical reimbursement records housed in the MHS Data Repository (MDR). The MDR contains health care data from TRICARE (ie, the military health care program) or TRICARE-reimbursed facilities, which include both military and civilian treatment facilities. The medical data captured reflects services used that were reimbursed by TRICARE, whether elective or mandated by a service member’s command (eg, command-directed substance use treatment). Therefore, MDR data represent the *use* of health care services, but not necessarily a *preference* for the care received.

Behavioral health diagnoses were identified based on records containing both (1) *International Classification of Diseases, 10th Revision* (ICD-10) [54], codes denoting conditions of interest; and (2) corresponding Current Procedural Terminology or Healthcare Common Procedure Coding System codes indicating the treatment modality (telehealth vs in-person) of each health care visit. Visits were only included if behavioral health treatment was provided for at least one of the eligible diagnoses. Behavioral health treatment included services such as individual psychotherapy, family or group therapy, diagnostic or psychological testing, health behavior interventions, psychiatry evaluation and management, and substance use treatment and intervention. Behavioral health visits were further classified as either in person or telehealth using relevant Current Procedural Terminology and Healthcare Common Procedure Coding System codes [8]. For example, 99443 designates a telephone evaluation or management visit lasting 21 to 30 minutes, and modifier code “95” denotes a synchronous audio-video visit delivered to a patient not located at a military treatment facility. These codes can be found in Multimedia Appendix 1.

For each service member in the sample, the total number of behavioral health visits, the modality of each visit, and the average duration of time between visits were calculated. Demographic data included sex, race and ethnicity, service branch, age, and rank at the first behavioral health visit.

### Ethical Considerations

The study protocol was approved by the Naval Health Research Center Institutional Review Board (NHRC.2022.0005) in compliance with all applicable federal regulations. This study used archival data, and therefore, informed consent and compensation were not part of the study. Data were accessed and protected following federal and US Department of Defense regulations.

### Statistical Analyses

Descriptive statistics were computed for all demographic variables, behavioral health conditions, and frequency of visits (total number of visits and average time between visits) for the full sample and then separately for each of the 3 care modality groups (ie, telehealth, in-person, and hybrid care). Chi-square tests of independence were used to assess the demographic distribution across treatment delivery modalities. As most demographic characteristics were categorical, post hoc tests were used to identify differences in treatment delivery modality against a reference group within each categorical demographic variable. Reference groups included non-Hispanic White race and ethnicity, the Marine Corps service branch, and junior enlisted rank. Cramer *V* statistics were then computed to determine the effect size of differences (with a large effect size defined as a Cramer *V* value of ≥0.15) between these groups.

Chi-square tests of equal proportions were used to assess statistical significance in the distribution of care across treatment delivery modalities for each of the 12 behavioral health conditions of interest. As the use of telehealth alone was less frequent than only in-person care or the hybrid of telehealth and in-person care during the observation period, post hoc tests were run to determine differences between in-person and hybrid care.

ANOVA tests were computed to assess differences in the average number of visits and the average time between visits across the 3 treatment delivery modality groups, both for care overall and for each specific behavioral health condition. An η^2^ statistic assessed the effect size of the results. In cases where the effect size was medium (0.06‐0.13) or large (≥0.14), a post hoc Tukey test was conducted to identify differences between the 3 treatment delivery modality groups for each behavioral health condition.

## Results

### Demographics

A total of 622,452 service members received care for the behavioral health conditions of interest during the study period and had at least 6 months of continuous service. After removing those with incomplete personnel records (n=33,524, 5.39%), the study sample consisted of 588,928 service members. Overall, 350,843 (59.57%) service members received only in-person care during the study period, 24,245 (4.12%) received only telehealth, and 213,840 (36.31%) received a hybrid of in-person and telehealth care.

Both omnibus and subsequent post hoc chi-square tests of independence revealed statistically significant differences in care use within each demographic variable (Table 1). However, no calculations produced a large effect size. Analyses between service branches, ranks, and sexes produced small effect sizes (Cramer *V* between 0.04 and 0.09). Specifically, compared with Marines, soldiers and airmen were more likely to use hybrid care, and those in the Coast Guard were more likely to use telehealth care alone. Compared with junior enlisted members, senior enlisted members, officers, and warrant officers were more likely to use telehealth care, both on its own and in conjunction with in-person care. Women were more likely to use a combination of in-person and telehealth services compared with men, who were more likely to use in-person services alone.

**Table 1.** Patient demographics by treatment modality.

Characteristics	Overall	Breakdown by treatment modality			Post hoc chi-square tests^a^	
		Both in-person and telehealth	In person only	Telehealth only	*P* value	Cramer *V*
Sex, n (%)						
Female	138,508 (23.60)	56,896 (41.08)	75,998 (54.87)	5614 (4.05)	<.01	0.05
Male	448,335 (76.40)	156,825 (34.98)	272,909 (60.87)	18,601 (4.15)	Reference	—^b^
Unknown	2085	—	—	—	—	—
Race and ethnicity, n (%)						
American Indian–Alaskan Native	16,947 (2.95)	6321 (37.30)	9979 (58.88)	647 (3.82)	<.01	0.01
Asian American–Pacific Islander	25,561 (4.45)	8806 (34.45)	15,593 (61.00)	1162 (4.55)	<.01	0.01
Black–African American	168,480 (29.32)	64,179 (38.09)	97,282 (57.74)	7019 (4.17)	<.01	0.03
Hispanic-Latino	56,647 (9.86)	19,650 (34.69)	34,655 (61.18)	2342 (4.13)	.02	0.01
Multiracial	38,281 (6.66)	15,355 (40.11)	21,374 (55.83)	1552 (4.05)	<.01	0.03
Non-Hispanic White	268,645 (46.76)	94,844 (35.30)	163,066 (60.70)	10,735 (4.00)	Reference	—
Unknown	14,367	—	—	—	—	—
Service branch, n (%)						
Air Force	121,008 (20.61)	46,615 (38.52)	68,601 (56.69)	5792 (4.79)	<.01	0.09
Army	268,136 (45.68)	104,241 (38.88)	154,841 (57.75)	9054 (3.38)	<.01	0.07
Coast Guard	9228 (1.57)	2255 (24.44)	6276 (68.01)	697 (7.55)	<.01	0.06
Marine Corps	64,288 (10.95)	19,223 (29.90)	42,295 (65.79)	2770 (4.31)	Reference	—
Navy	124,366 (21.19)	41,446 (33.33)	77,006 (61.92)	5914 (4.76)	<.01	0.04
Unknown	1902	—	—	—	—	—
Rank, n (%)						
Junior enlisted	250,207 (42.49)	82,039 (32.79)	160,157 (64.01)	8011 (3.20)	Reference	—
Officer or warrant officer	64,066 (10.88)	23,917 (37.33)	35,859 (55.97)	4290 (6.70)	<.01	0.09
Senior enlisted	274,655 (46.64)	107,884 (39.28)	154,827 (56.37)	11,944 (4.35)	<.01	0.08
Age at first visit (y), mean (SD)	28.48 (7.78)	28.89 (7.59)	28.10 (7.84)	30.38 (8.06)	<.01^c^	0.00

a These tests analyze the distribution of care modalities against a reference group within each categorical variable (ie, non-Hispanic White race or ethnicity, Marine Corps service branch, and junior enlisted rank).

b Not available.

c As a continuous variable, age distribution was assessed using a 1-way ANOVA test and corresponding η2 value.

### Care Delivery Modality by Diagnosis

Overall, and irrespective of delivery modality, service members most often received care for adjustment disorder (336,766/588,928, 57%) and insomnia (240,776/588,928, 41%), after which there was a steep drop-off (the next most prevalent condition was AUD 96,509/588,928, 16%; Table 2). Service members infrequently received care for panic disorder (3%), suicidal behavior (0.4%), and suicidal ideation (0.5%).

**Table 2.** Patient treatment modality by behavioral health diagnosis.

Diagnosis	Overall, n (%)^a^	Breakdown by treatment modality, n (%)			Post hoc chi-square tests^b^^c^^d^ (*P* value)
		Both in-person and telehealth	In person only	Telehealth only	
Acute stress disorder	25,304 (4.30)	10,632 (42.02)	13,855 (54.75)	817 (3.23)	<.01
Adjustment disorder	336,766 (57.18)	140,783 (41.80)	186,809 (55.47)	9174 (2.72)	<.01
Alcohol use disorder	96,509 (16.39)	49,339 (51.12)	45,548 (47.20)	1622 (1.68)	<.01
Attention-deficit/hyperactivity disorder	52,337 (8.89)	31,990 (61.12)	19,308 (36.89)	1039 (1.99)	<.01
Generalized anxiety disorder	59,046 (10.03)	34,289 (58.07)	23,158 (39.22)	1599 (2.71)	<.01
Insomnia	240,776 (40.88)	100,936 (41.92)	130,585 (54.24)	9255 (3.84)	<.01
Major depressive disorder	76,641 (13.01)	46,283 (60.39)	28,935 (37.75)	1423 (1.86)	<.01
Panic disorder	15,434 (2.62)	8503 (55.09)	6590 (42.70)	341 (2.21)	<.01
Posttraumatic stress disorder	82,517 (14.01)	49,269 (59.71)	31,639 (38.34)	1609 (1.95)	<.01
Substance use disorder	21,171 (3.76)	12,189 (54.98)	9648 (43.52)	334 (1.51)	<.01
Suicidal behavior	2350 (0.40)	1366 (58.13)	976 (41.53)	8 (0.34)	<.01
Suicidal ideation	2912 (0.49)	1327 (45.57)	1530 (52.54)	55 (1.89)	<.01

a The columns add up to a number higher than the total N because many people in the study population had more than one diagnosis.

b These post hoc chi-square tests of equal proportion were conducted between in-person care only and combination in-person and telehealth care.

c Analyses link behavioral health diagnosis to the visit modality.

d The unit of measurement is the patient, not the visit. In this analysis, we are investigating patients’ treatment modality overall, rather than the total number of visits administered.

Those with ASD, adjustment disorder, insomnia, and suicidal ideation had among the lowest use of hybrid telehealth and in-person care (42%‐46%), and the highest use of in-person care alone (approximately 55%). Service members with ADHD, GAD, MDD, PTSD, and suicidal behavior were most likely to use a hybrid of in-person and telehealth care (57%‐61%) and least likely to use in-person care alone (37%‐42%). Those with AUD, ADHD, PTSD, SUD, MDD, suicidal ideation, and suicidal behavior were the least likely to use telehealth services alone (0.34%‐2%). All post hoc tests revealed statistically significant differences in the use of in-person alone versus hybrid care for each behavioral health condition (*P<*.01).

### Number of Visits

On average, service members attended 10 visits during the study period (Table 3). Broken down by modality group, those using hybrid care averaged approximately 8 times the number of visits as those using only telehealth and approximately 3 times the number of visits as those receiving only in-person care (19, 2, and 6 visits, respectively).

**Table 3.** Number of visits by treatment modality and behavioral health diagnosis.

Diagnosis	Visits, mean (SD)	Breakdown by treatment modality, mean (SD)			ANOVA	
		Both in-person and telehealth	In person only	Telehealth only	*P* value	η^2^
All diagnoses	10.81 (13.79)	19.74 (16.57)	5.94 (8.58)	2.43 (4.03)	<.01	0.24
Acute stress disorder	2.09 (3.14)	2.55 (3.82)	1.78 (2.49)	1.38 (2.35)	<.01	0.02
Adjustment disorder	6.67 (8.50)	9.92 (10.49)	4.43 (5.71)	2.53 (3.92)	<.01	0.11
Alcohol use disorder	14.26 (15.54)	18.51 (16.71)	10.10 (12.92)	1.93 (2.88)	<.01	0.08
Attention-deficit/hyperactivity disorder	8.27 (8.76)	10.93 (9.63)	4.18 (4.86)	2.27 (2.62)	<.01	0.15
Generalized anxiety disorder	6.67 (9.04)	8.27 (10.29)	4.43 (6.28)	4.58 (6.58)	<.01	0.04
Insomnia	3.43 (4.31)	4.83 (5.46)	2.49 (2.89)	1.42 (1.22)	<.01	0.08
Major depressive disorder	8.32 (11.01)	10.41 (12.38)	5.17 (7.50)	4.28 (5.78)	<.01	0.06
Panic disorder	4.42 (6.57)	5.53 (7.68)	3.04 (4.40)	3.50 (6.40)	<.01	0.04
Posttraumatic stress disorder	12.02 (13.94)	15.35 (15.40)	7.23 (9.60)	4.15 (6.44)	<.01	0.09
Substance use disorder	8.95 (11.01)	11.50 (12.36)	5.98 (8.16)	1.77 (1.89)	<.01	0.07
Suicidal behavior	1.76 (2.11)	1.91 (2.37)	1.56 (1.68)	1.00 (0^a^)	<.01	0.01
Suicidal ideation	2.47 (3.28)	2.85 (3.89)	2.18 (2.66)	1.55 (1.02)	<.01	0.01

a SD=0, as all 8 patients attended 1 visit related to suicidal behavior.

The conditions associated with the highest average number of visits included AUD (average of 14 visits; *SD* =15.54), PTSD (average of 12 visits; *SD* =13.94), as well as ADHD, MDD, and SUD (average of 8‐9 visits; *SDs* =8.76-11/01). Insomnia, suicidal behavior, suicidal ideation, and ASD showed the fewest number of visits (2‐3 on average; *SDs* =2.11-4.31). Although all ANOVA tests indicated statistically significant differences in the distribution of care modality within each condition, only 7 of the 12 conditions demonstrated a medium or large effect size (as defined by an η^2^ statistic between 0.06 and 0.13 and ≥0.14, respectively). Those seeking care for adjustment disorder, AUD, ADHD, insomnia, MDD, PTSD, and SUD were more likely to receive hybrid care than either in-person or telehealth care alone, as indicated by η^2^ statistics and subsequent Tukey tests.

### Time Interval Between Visits

Overall, the average interval between behavioral health visits was 67 days (*SD* =138.81; Table 4). In-person–only visits had the longest intervals (70 d; *SD* =153.95), followed by hybrid visits (64 d; *SD* =120.00) and telehealth visits (59 d; *SD* =139.18). Across specific behavioral health conditions, average intervals were longest among those with ASD, ADHD, and insomnia (79‐89 d; *SDs* =116.65-166.60), and the shortest among those with AUD, SUD, and suicidal behavior (25‐38 d; *SDs* =60.93-99.85). In all but 3 conditions (AUD, ADHD, and SUD), those who received only in-person care had the longest average intervals between visits. In 6 of the 12 conditions (ASD, AUD, ADHD, insomnia, PTSD, and SUD), those who received hybrid care of both in-person and telehealth had the shortest interval between visits. However, no models produced a medium or large effect size. Suicidal behavior did not present a significant difference between modalities.

**Table 4.** Number of days between visits by treatment modality and behavioral health diagnosis.

Diagnosis	Days between visits, mean (SD)	Breakdown by treatment modality, mean (SD)			ANOVA	
		Both in-person and telehealth	In person only	Telehealth only	*P* value	η^2^
All diagnoses	67.07 (138.81)	63.57 (120.00)	70.54 (153.95)	59.12 (139.18)	<.01	0.00
Acute stress disorder	79.05 (157.14)	68.09 (122.39)	91.71 (188.61)	74.80 (151.53)	<.01	0.01
Adjustment disorder	62.33 (127.20)	59.19 (107.54)	65.73 (144.06)	53.38 (131.39)	<.01	0.00
Alcohol use disorder	38.77 (99.85)	33.28 (76.34)	45.89 (123.29)	59.84 (169.24)	<.01	0.00
Attention-deficit/hyperactivity disorder	80.48 (116.65)	75.92 (97.60)	89.46 (146.93)	98.13 (163.71)	<.01	0.00
Generalized anxiety disorder	65.39 (118.25)	58.24 (98.66)	79.27 (146.02)	38.19 (92.95)	<.01	0.01
Insomnia	89.53 (166.60)	75.66 (137.03)	105.31 (193.44)	100.51 (194.46)	<.01	0.01
Major depressive disorder	50.99 (94.97)	45.45 (77.49)	61.68 (120.21)	35.35 (79.00)	<.01	0.01
Panic disorder	64.65 (125.36)	55.19 (95.16)	80.45 (161.05)	45.42 (128.93)	<.01	0.01
Posttraumatic stress disorder	52.92 (104.80)	47.05 (83.30)	63.53 (133.41)	48.34 (133.14)	<.01	0.01
Substance use disorder	30.12 (72.07)	26.66 (58.88)	35.34 (88.21)	42.69 (96.45)	<.01	0.00
Suicidal behavior	25.16 (60.93)	22.96 (32.48)	29.02 (91.40)	22.25 (5.30)	.09	0.00
Suicidal ideation	59.61 (108.08)	50.36 (76.22)	70.47 (134.66)	25.01 (40.29)	<.01	0.01

## Discussion

### Principal Findings

This study explored the modality of behavioral health care—telehealth, in-person, and hybrid care—delivered to active duty service members within the MHS from 2015 to 2021. This 6-year period spanned from when telehealth was seldom used before the COVID-19 pandemic [7,51,52], during the pandemic when restrictions led to a surge in telehealth use, and after the most stringent pandemic-related restrictions were lifted. During the study period, most service members (60%) received only in-person care, a sizable minority (36%) received a hybrid of telehealth and in-person care, and few (4%) received only telehealth. The higher proportion of in-person and hybrid care may be influenced by the observation period, which consisted of mostly prepandemic years when telehealth was seldom used within the MHS [7,51,52]. The modality of behavioral health care was also examined by demographic characteristics and behavioral health diagnoses. Significant demographic differences emerged showing that women were more likely to use a hybrid of in-person and telehealth care, whereas men more frequently used in-person services alone. Soldiers and airmen used hybrid care more often compared with Marines, while those in the Coast Guard more commonly used telehealth alone. Finally, officers, warrant officers, and senior enlisted members were more likely than junior-enlisted members to use telehealth, both on its own and in combination with in-person care.

Regarding behavioral health diagnoses, service members with ASD, adjustment disorder, insomnia, and suicidal ideation had the highest use of only in-person care and the lowest use of hybrid care. Service members with ADHD, GAD, MDD, PTSD, and suicidal behavior were most likely to use hybrid care and least likely to use only in-person care. Those with AUD, SUD, ADHD, PTSD, MDD, suicidal ideation, and suicidal behavior were least likely to use only telehealth. For 8 of the 12 behavioral health conditions of interest (AUD, ADHD, GAD, MDD, panic disorder, PTSD, SUD, and suicidal behavior), service members were more likely to receive hybrid care, whereas the other 4 conditions (ASD, adjustment disorder, insomnia, and suicidal ideation) were more likely to be associated with in-person care. Although these demographic and diagnostic findings were statistically significant and showed patterns of behavioral health care delivery, effect sizes were small.

Study analyses also compared the delivery modalities in terms of number of visits and between-visit intervals as care metrics. Service members using hybrid care averaged approximately 8 times the number of visits as those using only telehealth and 3 times the number of visits as those receiving in-person care. Specifically, service members who received hybrid care averaged 19 visits (*SD* =16.57), those who received in-person care only averaged 6 visits (*SD* =8.58), and those who received telehealth care only averaged 2 visits (*SD* =4.03). While a sufficient dose of psychotherapy can range depending on clinical factors, such as symptom severity or comorbidities, even a minimally sufficient dose of 9 sessions (such as for PTSD) [55], suggests that only the hybrid group met this threshold.

The average between-visit interval across behavioral health diagnoses was 67 days. This interval exceeds the recommended and commonly evaluated frequencies of once or twice weekly sessions for cognitive behavioral therapies [56,57]. Longer time between sessions was associated with increased dropout among service members in treatment for PTSD [58]. However, it should be noted that not all behavioral health visits were for psychotherapy, and some conditions (eg, ADHD) may be successfully treated with fewer sessions of medication management. Further exploring the frequency of behavioral health care use within the MHS is critical, as it could significantly affect service members’ behavioral health and operational readiness through relevant behavioral health policy.

For between-visit intervals across delivery modalities, this difference was statistically significant and amounted to approximately 11 days (70 for in-person only vs 59 for telehealth only). However, it is difficult to determine the extent to which this duration is clinically meaningful. For between-visit intervals across diagnoses, except for 3 (AUD, ADHD, and SUD), service members who received only in-person care had the longest average intervals between visits. Conversely, in 6 of the 12 conditions (ASD, AUD, ADHD, insomnia, PTSD, and SUD), those who received a hybrid of both in-person and telehealth care had the shortest interval between visits. Among specific behavioral health conditions, the longest average intervals were among service members with ASD, ADHD, and insomnia. The shortest intervals were observed among service members with suicidal behavior, SUD, and AUD, which aligns with clinical necessity, as these are presenting concerns often requiring urgent care due to safety risks. One condition, suicidal behavior, did not have a significant difference in the length between visits among the treatment modalities. This may be due, in part, to the small sample size of those with suicidal behavior; for example, there were 8 service members who received only telehealth care.

### Comparison With Prior Work

Prior research revealed increased telehealth care use in both the MHS and civilian hospital settings immediately following the onset of the COVID-19 pandemic [4-7,32]. However, the use of a hybrid of telehealth and in-person care has seldom been explored. In a study that examined modalities of treatment delivery, most service members with PTSD, depression, or SUD who initiated behavioral health care early in the pandemic received a hybrid of telehealth and in-person visits [8].

This study adds to the existing literature in several ways. First, the use of telehealth, in-person, and hybrid care was explored over a 6-year period ending in September 2021. In contrast, Hepner et al [8] used corresponding 6-month observation periods (April to September) in 2019 and 2020. The selected time points between these 2 studies highlight different aspects of the data. For example, during this 6-year observation period that included years before the COVID-19 pandemic, in-person behavioral health care was the most common mode of treatment delivery (60%), whereas in the early months following the onset of the pandemic, a hybrid mode of delivery was most frequently received (50%‐56%) [8]. Second, this study uniquely explored whether treatment delivery modality differed across 12 behavioral health conditions of interest. This research question is distinct from that addressed by Hepner et al [8], which determined visits by PTSD, depression, and SUD diagnoses between pre- and post-pandemic periods.

Finally, this work explored care metrics through the number of visits received and intervals between visits across behavioral health conditions and by delivery modality, which showed both similarities and differences with existing research. This study showed similar findings to those of Cozzens [31] regarding reduced time to access care for telehealth compared with in-person visits. The care metrics in this study varied from those explored by Hepner et al [8], which focused on treatment initiation and transitions of care by the 3 diagnoses of interest during the pre- and post-pandemic periods rather than by both diagnosis and delivery modality. In sum, this study complements and builds on the existing literature by extending the postpandemic period and determining the use of delivery modality across a wide array of behavioral health diagnoses, which can inform ongoing health care delivery within the MHS.

### Limitations

There are several limitations that should be considered when interpreting the results of this research. Study data were not based on gold standard, diagnostic assessments but rather, were derived from diagnostic and procedural codes documented in electronic medical records, which may be subject to factors such as coding errors, provider knowledge, and the extent of symptoms discussed in an appointment. Specific to telehealth, there was evolving guidance regarding how providers should code for telehealth services in the MHS that could have contributed to variability [8]. Although guidance issued directly to behavioral health providers during the pandemic period was obtained by the authors and reviewed for the extraction of relevant codes, the validity of these codes over time cannot be ascertained. Health service and policy researchers have proposed guidance for providers regarding the coding of telehealth services in the MHS to improve data accuracy [8,31]. Additionally, data were only available for service members who received behavioral health care that was reimbursed by TRICARE, and findings may not extend beyond this population. Separate courses of treatment could not be determined from medical record data and, with the 6-year period, may result in longer average between-visit intervals. Data from medical records indicate health care use and may not represent the care preferences of service members or satisfaction with care received. Finally, this study captured trends over an observation period that included a critical period in telehealth use within the MHS; however, it does not reflect current patterns of modality use or those since the declassification of the COVID-19 pandemic as a public health emergency [59], thus necessitating ongoing research efforts.

### Conclusions

Behavioral health conditions can adversely affect service members and operational readiness. Offering options beyond in-person behavioral health care may improve access to care, as study results demonstrated. Collectively, findings from the 6-year observation period showed that telehealth was commonly used in combination with in-person care. Furthermore, telehealth was related to more behavioral health care visits and the least amount of time between visits, highlighting the advantages of offering telehealth as an option within the MHS health care landscape. Although study findings support telehealth as an option for treatment delivery, it may not be ideally suited for all service members or in all situations [32], and in-person or hybrid care delivery may be preferred by a patient or deemed more clinically appropriate by a provider. Given options for care delivery within the MHS, it is recommended that treatment modality be selected based on patient preference and shared decision-making [49,50]. Additionally, providing ongoing flexibility, regularly reassessing preferences, and personalizing treatment are important aspects for the delivery of optimal behavioral health care to service members [22,50], along with the infrastructure and policies to support these practices [42]. This study contributes novel information about behavioral health treatment delivery within the MHS, but further research is needed to explore service member preferences for delivery modality (telehealth, in-person, and hybrid care) and how preferences align with care received and treatment outcomes.

## Supplementary material

10.2196/83809 Multimedia Appendix 1 Medical billing codes denoting behavioral health treatment, telehealth treatment, and behavioral health diagnosis.

## Abbreviations

ADHD: attention-deficit/hyperactivity disorder
ASD: acute stress disorder
AUD: alcohol use disorder
GAD: generalized anxiety disorder
ICD-10: International Classification of Diseases, 10th Revision
MDD: major depressive disorder
MDR: Military Health System Data Repository
MHS: Military Health System
PTSD: posttraumatic stress disorder
SUD: substance use disorder
