# Supplementary material for: Telebehavioral Health, In-Person, and Hybrid Modalities of Treatment Delivery Among US Service Members: Longitudinal Observational Study
Source: JMIR Ment Health. 2026 Jan 5;13:e83809. doi: 10.2196/83809 (PMC12768398; doi:10.2196/83809)
Supplement: Multimedia Appendix 1 [file mental-v13-e83809-s001.docx]

**Table S1.** Medical billing codes denoting behavioral health treatment, telehealth treatment, and behavioral health diagnosis.

| CPT codes (behavioral health treatment) |  |
| --- | --- |
| Current Procedural Terminology | 90791, 90792, 90832-90834, 90836-90838, 90845-90847, 90849, 90853, 90839, 90840, 96105, 96112, 96113, 96116, 96121, 96125, 96127, 96130-96133, 96136-96139, 96156, 96158-96160, 96164, 96165, 96167, 96167, 96168, 96170, 96171, 98966-98969, 99201-99205, 99211-99215, 99366, 99367, 99406-99409, 99242-99245, 99499, 99441-99443, 99446-99449 |
| CPT or HCPCS codes (telehealth treatment) |  |
| Current Procedural Terminology | 99441-99449, 96160, 98966-98969, modifier 95 |
| Healthcare Common Procedure Coding System | G2012, T2025, Q3014, modifier GT or GQ |
| ICD-10 codes (behavioral health diagnosis) |  |
| Acute stress disorder | F430 |
| Adjustment disorder | F4321, F4322, F4323, F4324, F4325, F4320 |
| Alcohol use disorder | F1010, F1011, F1011, F1020, F1021, F1021, F1020, F10120, F10220, F10920, F10130, F10230, F10930, F10132, F10232, F10932, F10159, F10259, F10959, F1014, F1024, F1094, F10180, F10280, F10980, F10182, F10282, F10982, F10181, F10281, F10981, F10121, F10221, F10921, F10131, F10231, F10931, F1026, F1096, F1027, F1097, F10188, F10288, F10988, F1099 |
| Attention-deficit/hyperactivity disorder | F902, F900, F901, F908, F909 |
| Generalized anxiety disorder | F411 |
| Insomnia | F5101, F5102, F5103, F5104, F5105, F5109, G4709, G4700 |
| Major depressive disorder | F330, F331, F332, F333, F3341, F3342, F339 |
| Panic disorder | F410 |
| Posttraumatic stress disorder | F4310, F4311, F4312 |
| Substance use disorder | F1210, F1211, F1211, F1220, F1221, F1221, F1220, F12120, F12220, F12920, F12122, F12222, F12922, F1213, F1223, F1293, F12159, F12259, F12959, F12180, F12280, F12980, F12188, F12288, F12988, F12121, F12221, F12921, F12921, F1299, F1610, F1611, F1611, F1620, F1621, F1621, F1620, F16120, F16220, F16920, F16983, F16159, F16259, F16959, F1614, F1624, F1694, F16180, F16280, F16980, F16121, F16221, F16921, F16921, F1699, F1699, F1810, F1811, F1811, F1820, F1821, F1821, F1820, F18120, F18220, F18920, F18159, F18259, F18959, F1814, F1824, F1894, F18180, F18280, F18980, F18121, F18221, F18921, F1817, F1827, F1897, F18188, F18288, F18988, F1899, F1110, F1111, F1111, F1120, F1121, F1121, F1120, F11120, F11220, F11920, F11122, F11222, F11922, F1113, F1123, F1193, F1114, F1124, F1194, F11188, F11288, F11988, F11182, F11982, F11181, F11281, F11981, F11121, F11221, F11921, F11921, F11988, F1199, F1310, F1311, F1311, F1320, F1321, F1321, F1320, F13120, F13220, F13920, F13130, F13230, F13930, F13132, F13232, F13932, F13159, F13259, F13959, F1314, F1324, F1394, F13180, F13280, F13980, F13182, F13282, F13982, F13181, F13281, F13981, F13121, F13221, F13921, F13131, F13231, F13931, F13921, F13931, F1327, F1397, F13188, F13288, F13988, F1399, F1510, F1410, F1510, F1511, F1411, F1511, F1520, F1420, F1520, F1521, F1421, F1521, F15120, F15220, F15920, F14120, F14220, F14920, F15122, F15222, F15922, F14122, F14222, F14922, F1513, F1523, F1593, F1413, F1423, F1493, F15159, F15259, F15959, F14159, F14259, F14959, F1514, F1524, F1594, F1414, F1424, F1494, F15180, F15280, F15980, F14180, F14280, F14980, F15188, F15288, F15988, F14188, F14288, F14988, F15182, F15282, F15982, F14182, F14282, F14982, F15181, F15281, F15981, F14181, F14281, F14981, F15121, F15221, F15921, F14121, F14221, F14921, F15921, F1599, F1499 |
| Suicidal behavior | T1491A, T1491D |
| Suicidal ideation | R4581 |

ICD-10, International Classification of Diseases, Tenth Revision.

**Reference**

Centers for Medicare & Medicaid Services. List of CPT/HCPCS Codes. 2025. https://www.cms.gov/medicare/regulations-guidance/physician-self-referral/list-cpt-hcpcs-codes [accessed 2024-11-15]
